# Protection of Spanish Ibex (*Capra pyrenaica*) against Bluetongue Virus Serotypes 1 and 8 in a Subclinical Experimental Infection

**DOI:** 10.1371/journal.pone.0036380

**Published:** 2012-05-30

**Authors:** Cristina Lorca-Oró, Joan Pujols, Ignacio García-Bocanegra, Gregorio Mentaberre, José Enrique Granados, David Solanes, Paulino Fandos, Iván Galindo, Mariano Domingo, Santiago Lavín, Jorge Ramón López-Olvera

**Affiliations:** 1 Centre de Recerca en Sanitat Animal (CReSA), UAB-IRTA, Campus de la Universitat Autònoma de Barcelona, Barcelona, Spain; 2 Institut de Recerca i Tecnologia Agroalimentàries (IRTA), Barcelona, Spain; 3 Departamento de Sanidad Animal. Facultad de Veterinaria, UCO, Campus Universitarios de Rabanales, Córdoba, Spain; 4 Servei d'Ecopatologia de Fauna Salvatge (SEFaS), Departament de Medicina i Cirurgia Animals, Universitat Autònoma de Barcelona (UAB), Barcelona, Spain; 5 Parque Nacional y Parque Natural de Sierra Nevada, Carretera Antigua de Sierra Nevada, Pinos Genil, Granada, Spain; 6 Agencia de Medio Ambiente y Agua, Granada, Spain; 7 Agencia de Medio Ambiente y Agua, Isla de la Cartuja, Sevilla, Spain; 8 Departament de Sanitat i Anatomia Animals, Universitat Autònoma de Barcelona, Barcelona, Spain; Friedrich-Loeffler-Institut, Germany

## Abstract

Many wild ruminants such as Spanish ibex (*Capra pyrenaica*) are susceptible to Bluetongue virus (BTV) infection, which causes disease mainly in domestic sheep and cattle. Outbreaks involving either BTV serotypes 1 (BTV-1) and 8 (BTV-8) are currently challenging Europe. Inclusion of wildlife vaccination among BTV control measures should be considered in certain species. In the present study, four out of fifteen seronegative Spanish ibexes were immunized with a single dose of inactivated vaccine against BTV-1, four against BTV-8 and seven ibexes were non vaccinated controls. Seven ibexes (four vaccinated and three controls) were inoculated with each BTV serotype. Antibody and IFN-gamma responses were evaluated until 28 days after inoculation (dpi). The vaccinated ibexes showed significant (*P*<0.05) neutralizing antibody levels after vaccination compared to non vaccinated ibexes. The non vaccinated ibexes remained seronegative until challenge and showed neutralizing antibodies from 7 dpi. BTV RNA was detected in the blood of non vaccinated ibexes from 2 to the end of the study (28 dpi) and in target tissue samples obtained at necropsy (8 and 28 dpi). BTV-1 was successfully isolated on cell culture from blood and target tissues of non vaccinated ibexes. Clinical signs were unapparent and no gross lesions were found at necropsy. Our results show for the first time that Spanish ibex is susceptible and asymptomatic to BTV infection and also that a single dose of vaccine prevents viraemia against BTV-1 and BTV-8 replication.

## Introduction

Domestic and wild ruminants are thought to be susceptible to bluetongue virus (BTV) infection, which causes bluetongue (BT), a disease that has a high economic impact on animal health. BTV belongs to the genus *Orbivirus* (family Reoviridae) and is transmitted by blood-feeding midges of the genus *Culicoides* (*Diptera*, *Ceratopogonidae*) [1], [2]. There are at least 24 different BTV serotypes, and two putative new serotypes, the 25th named Toggenburg orbivirus [3], [4] and a 26th [5], coinciding with the distribution of competent vectors in all continents except Antarctica. BT is considered an emerging and re-emerging disease in Europe. Since 1998, at least eight serotypes (BTV-1, -2, -4, -6, -8, -9, -11 and -16) have been detected in Europe, where BT has expanded its geographical range northwards [6]–[9].

The spreading of BTV-8 through Europe since its introduction in 2006 caused severe disease, mainly in cattle, but also in sheep, and heavy financial losses in animal industry. Previously, BTV-1 infections caused epizootics in southern Europe. BTV-1 and -8 were detected in livestock in Spain in 2007 and 2008, respectively. For safety reasons, immunization against BTV-1 was carried out together with a mass vaccination campaign against BTV-8 using inactivated vaccines to control the expansion of these serotypes in the affected countries of Europe. The target of the vaccination program was to achieve at least 80% coverage of susceptible ruminants [6], [8], [10].

The origin of BT is probably African, and wild ruminants are the natural hosts of BTV, although it is thought that cattle have replaced antelope as BTV maintenance host [11]. Information on the role of wild ruminants in the maintenance and spread of BTV is still limited. Several studies have been performed in wild ruminants from North America, where a range of species are frequently infected with BTV [12]. However, studies on the susceptibility of native wild ruminant species are scarce in Europe. From 2006 to 2010, antibodies against BTV-1, -4, and -8 have been found in red deer (*Cervus elaphus*), fallow deer (*Dama dama*), mouflon (*Ovis aries musimon*), roe deer (*Capreolus capreolus*), aoudad (*Amotragus lervia*) and Spanish ibex (*Capra pyrenaica*) in Spain [13]–[19]. Although BTV infection is often subclinical or unapparent in some wild ruminants, bighorn sheep (*Ovis canadiensis*) and mouflon can develop fatal clinical disease, as do closely related domestic sheep [17], [20], [21]. Experimental infection of pronghorn antelope (*Antilocapra americana*), American bison (*Bison bison*) and Affrican buffalo (*Syncerus caffer*) also produced clinical disease [22], [23], whereas blesbock (*Damaliscus pygarus*) [24] and mountain gazelle (*Gazella gazella*) [25] did not show clinical signs after natural or experimental infection. Recent studies observed susceptibility to experimental infection with BTV-8 in red deer [26]. Red deer vaccination against BTV-1 and BTV-8 has proved to be safe and effective to prevent viraemia in experimentally inoculated deer [27].

Spanish ibex is an endemic species from Spain, with populations widespread throughout the southern and eastern regions of the country [28]. This wild mountain ungulate has a great value for its conservation as it has been listed as threatened and currently of least concern in the IUCN Red List of Threatened Species [29]. In the last decades, contagious diseases such as sarcoptic mange, habitat fragmentation, illegal hunting, loss of genetic diversity, local overabundance and disequilibrium in the population sex ratio and age structure have also contributed to a significant decline of its populations [30]–[33]. Spanish ibex frequently share the same habitat with domestic ruminants, especially in summer months when exploiting the summer high mountain pastures [28]. Allochthonous wild ungulate species, such as aoudad, fallow deer and mouflon, also suppose a threat to Spanish ibex and increase the potential risk of shared diseases transmission [34]–[35].

The aim of the present study is to evaluate the efficacy of commercial inactivated BTV vaccines in Spanish ibex, a potential BTV susceptible species.

## Results

### Antibody response to vaccination and infection

**Figure 1:**
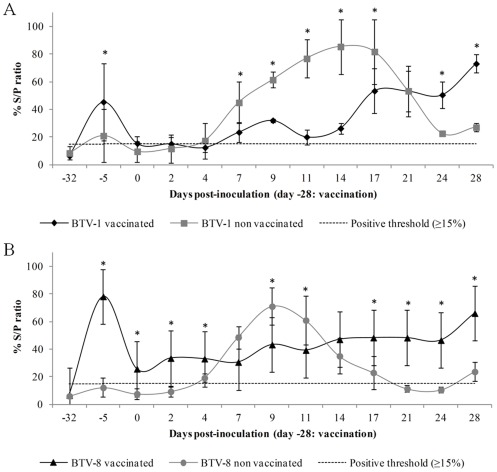
ELISA antibody response after BTV vaccination and experimental infection. Evolution of mean percentages (O.D. sample/O.D. positive control ×100) with standard deviation of VP7 antibodies for each group of vaccinated and non vaccinated ibexes challenged with BTV-1 (A) and BTV-8 serotypes (B).

Non vaccinated ibexes were seronegative until challenge. BTV-specific antibodies measured by ELISA increased significantly (*P*<0.05) by 23 days after vaccination (−5 dpi) in the vaccinated ibexes, which showed protective antibody levels along the challenge. Conversely, BTV antibodies increased from 4–7 dpi in the non vaccinated ibexes, reaching its maximum at 17 dpi for BTV-1, and at 9 dpi for BTV-8, which showed a shorter and faster dynamics than BTV-1. Mean and standard deviation of percentage values of VP7 ELISA assays before and after BTV challenge are shown in Figure 1.

**Figure 2:**
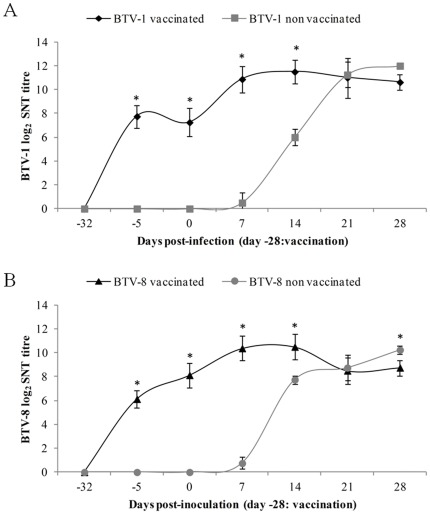
Neutralizing antibody response after BTV vaccination and experimental infection. Evolution of mean BTV-1 (A) and BTV-8 (B) neutralizing antibody titres (with standard deviation) for vaccinated and non vaccinated ibexes.

Neutralizing antibodies followed a similar pattern in both the BTV-1 and BTV-8 inoculated groups, either vaccinated or non vaccinated. The vaccinated ibexes showed statistically significant higher antibody titres (*P*<0.05) by serum neutralization (SNT) than the corresponding non vaccinated groups from −5 to 14 dpi. Non vaccinated ibexes started to show neutralizing antibodies from 7 dpi in both inoculated groups, reaching similar values to the vaccinated groups by 21 dpi (Figure 2).

### BTV RNA detection and isolation

**Figure 3:**
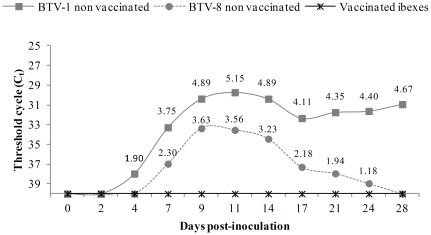
Detection of viraemia after BTV inoculation. Threshold cycle (*C_t_*) values of real-time RT-PCR from blood samples of vaccinated and non vaccinated ibexes challenged with BTV-1 and BTV-8. Negative results are shown as a *C_t_* of 40. Superscripts indicate the estimated BTV titres (TCID_50_/ml).

No BTV RNA was detected in any blood sample of the vaccinated ibexes during the experimental period. BTV-1 non vaccinated inoculated ibexes were RT-PCR positive from 4 (two out of three) or 7 (one out of three) dpi until 28 dpi. For the BTV-8 inoculated ibexes, one non vaccinated ibex was RT-PCR positive from 7 to 17 dpi, a second one from 9 to 14 dpi and the one euthanized at 8 dpi remained negative. Results of RT-qPCR are shown as *C_t_* values and estimated titres in Figure 3. BTV was successfully isolated in Vero cells only from blood samples of the two BTV-1 non vaccinated inoculated ibexes at 7 and 9 dpi. Blood samples from the BTV-8 non vaccinated and all the vaccinated ibexes were negative to virus isolation throughout the experimental period.

RT-qPCR results from the tissue samples of the three ibexes euthanized at 8 dpi are shown in Table 1. BTV was isolated in the BTV-1 non vaccinated inoculated ibex from spleen, lymph nodes (prescapular, axillary, ileal, gastric and submandibular), diaphragmatic lung lobe, nasal mucosae and pulmonary artery. BTV was not isolated from any sample of the BTV-8 inoculated ibex. At 28 dpi, all the four remaining inoculated and non vaccinated ibexes were RT-qPCR positive for spleen and lymph node samples as shown in Table 2. BTV was not isolated from any sample of any ibex at 28 dpi.

**Table 1:** Threshold cycle (*C_t_*) values and estimated titres (TCID_50_/ml) of specific real-time RT-PCR results on tissue samples at 8 dpi.

Tissue sample	BTV-1 non vac.		BTV-8 non vac.	
	Ct	TCID_50_/ml	Ct	TCID_50_/ml
**Spleen**	29.93	3.93	33.83	2.78
**Prescapular lymph node**	28.29	4.41	34.62	2.55
**Mediastinic lymph node**	31.57	3.45	36.29	2.05
**Axillary lymph node**	29.72	3.99	35.77	2.21
**Ileal lymph node**	30.41	3.79	35.69	2.23
**Gastric lymph node**	29.18	4.15	35.06	2.42
**Submandibular lymph node NODEnode**	30.09	3.88	35.22	2.37
**Apical lung lobe**	31.28	3.53	35.93	2.16
**Middle lung lobe**	30.09	3.88	34.69	2.53
**Diaphragmatic lung lobe**	30.41	3.79	34.91	2.46
**Liver**	28.19	4.44	32.70	3.11
**Kidney**	30.59	3.74	32.62	3.14
**Nasal mucosae**	32.02	3.31	36.54^b^	1.98
**Oral mucosae**	33.48	2.88	N	
**Lip**	34.97	2.44	N	
**Tongue**	33.39	2.91	37.69^b^	1.64
**Axillary skin**	N^a^		N	
**Palate**	34.80	2.49	N	
**Pulmonary artery**	32.47	3.18	37.94^b^	1.57
**Heart**	31.50	3.46	37.02^b^	1.84
**Epididymis**	32.00	3.32	37.01^b^	1.84
**Testicle**	30.66	3.72	36.34^b^	2.04
**Urinary bladder**	31.51	3.46	N	
**Ileum**	33.39^b^	2.91	N	
**Ileocaecal valve**	36.73	1.92	N	

a Sample positive to conventional RT-PCR.

b Sample negative to conventional RT-PCR.

Undetermined results (out of the detection level) are shown as Negative (N). Samples without superscript were coincident with conventional RT-PCR. The tissue samples from the non vaccinated and non inoculated ibex were all negative.

**Table 2:** Threshold cycle (*C_t_*) values and estimated titres (TCID_50_/ml) of specific real-time RT-PCR results on tissue samples at necropsy at the end of the study (28 dpi).

Treatment	Ibex num.	Tissue sample					
		Spleen		Prescapular lymph node		Mediastinic lymph node	
		Ct	TCID_50_/ml	Ct	TCID_50_/ml	Ct	TCID_50_/ml
**BTV-1 non vac.**							
	**215**	30.41	3.79	26.47	4.95	31.17	3.56
	**220**	30.60	3.73	28.24	4.43	30.96	3.63
**BTV-1 vac.**							
	**227**	N		35.80	2.20	N	
	**306**	N		N		N	
	**310**	N		N		N	
	**312**	N		N		N	
**BTV-8 non vac.**							
	**217**	30.52	3.76	31.65	3.42	33.71	2.81
	**307**	33.03	3.02	34.18	2.68	36.37	2.03
**BTV-8 vac.**							
	**225**	N		33.06	3.01	39.24	1.48
	**303**	N		37.55	1.68	N	
	**304**	34.70	2.52	29.75	3.98	N	
	**305**	N		31.05	3.60	N	

Undetermined results (out of the detection level) are shown as Negative (N).

### IFN-γ response in PBMCs

**Figure 4:**
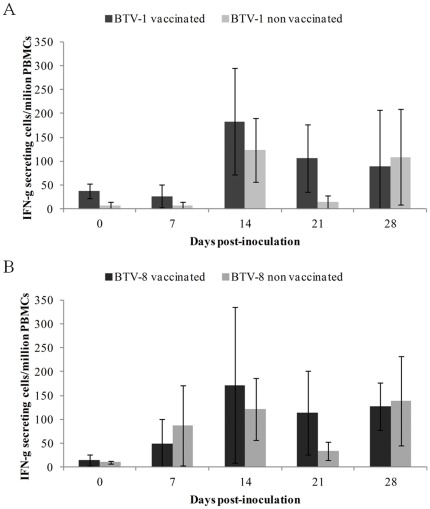
Interferon-gamma spot numbers produced by PBMCs after stimulation with the homologous strains. Mean with standard deviation of IFN-γ expression in 10^6^ PBMCs stimulated after challenge with BTV-1 (A) and BTV-8 (B). Counting of spots of the negative control has been substracted from each sample.

No statistically significant differences in IFN-γ response of stimulated PBMCs were found between vaccinated and non vaccinated ibexes. However, ELISPOT assays revealed that, after immunization and infection with BTV, the PBMCs reacted to the stimulation with the homologous strains of each serotype, especially at 14 and 21 dpi, showing an increase in the expression of IFN-γ. Countings of IFN-γ-SC are shown in Figure 4.

### Haematology

The BTV-1 inoculated and vaccinated ibexes showed statistically significant higher leukocyte (7, 11, and 14 dpi) and monocyte (7 and 11 dpi) counts than the BTV-1 inoculated non vaccinated ones.

Conversely to the BTV-1 inoculated ibexes, lymphocyte (4, 9, 17, and 24 dpi) and monocyte (11 dpi) counts were higher in the BTV-8 inoculated and non vaccinated ibexes than in the BTV-8 inoculated vaccinated ones.

No statistically significant differences between the vaccinated and non vaccinated groups were found for red blood cell count (RBC), platelet count (PLT), haemoglobin concentration (HGB), haematocrit (HTC), mean corpuscular volume (MCV), mean corpuscular haemoglobin (MCH) and mean corpuscular haemoglobin concentration (MCHC).

### Clinical signs and histopathological examination

No clear clinical signs compatible with BTV infection were detected throughout after inoculation (from 0 to 28 dpi). Only the non vaccinated ibexes inoculated with BTV-1 had a punctual increase (*P*>0.05) in rectal temperature at 7 dpi. No gross lesions were found after challenge, at necropsy, or after histological analysis of target tissues.

## Discussion

BTV RNA detection both by RT-PCR and RT-qPCR, and BTV isolation without specific clinical signs confirm the susceptibility of Spanish ibex to asymptomatic BTV infection. Overall, viraemia dynamics was rather similar to that of other asymptomatic hosts, either domestic (cattle and goat) or wild, namely red deer [26], [27], [36]–[38], than to that of clinically susceptible species, also either domestic (sheep) or wild (white-tailed deer and mouflon) [20], [38]–[41]. As demonstrated by ELISA and SNT, vaccination induced protective neutralizing antibodies against BTV-1 and BTV-8 in the vaccinated Spanish ibexes, which did not develop viraemia. Therefore, one only dose of vaccination protected Spanish ibex against BTV infection. This enhances the potential usefulness of wild ruminant vaccination as a complementary tool to control BTV transmission and permits a successful immunization with only one handling. After the antibody peak measured by ELISA in the non-vaccinated groups (at 14 dpi in BTV-1 and 9 dpi in BTV-8) antibody levels decreased, even until negative results in the BTV-8 group. These findings are in agreement with the previously observed in domestic ruminants by Echbaumer et al. 2011 [42], who demonstrate that double antigen ELISAs are highly sensitive for vaccine induced antibodies but might be less sensitive after infection. Also, in the neutralization test all non-vaccinated animals seroconverted and remained positive at high levels until the end of the experiment.

To the authors' knowledge, this is the first time that IFN-γ expression is studied in Spanish ibex as a first approach to the cellular immune response to BTV infection. IFN-γ is secreted by natural killer cells, CD4 and CD8 T cells as a response to BTV infection [43]. The increase in IFN-γ expression found in the vaccinated ibexes after BTV inoculation agrees with previous results in domestic sheep and cattle [44], [45]. Monocytes are a preferential target for BTV [46], [47]. Higher monocyte counts in the BTV-1 vaccinated ibexes at 7 and 11 dpi could mean that these animals were having a cellular response after the challenge. In spite of reported immune cross reaction between BTV-1 and BTV-8, the differences found in leukocyte, monocyte and lymphocyte trends between the BTV-1 and BTV-8 inoculated Spanish ibexes suggest differences between the strains used in this study (BTV-1/ALG/2006 and BTV-8/BEL/2006, respectively), not only in pathogenesis, but also in vaccine action pathways. Differences in pathogenesis regarding the same virus isolates (BTV-1 and BTV-8) in other wild ruminant species, namely red deer, have been previously suggested [27]. The more disseminated tissue distribution of BTV-1 as compared to BTV-8 at 8 dpi (Table 1) seems to further confirm this difference in pathogenesis between both strains. A similar low transient BTV-8 RNA detection after experimental infection has already been reported in red deer [26], [27]. The positive tissue samples from vaccinated ibexes found at 28 dpi without showing viraemia or seroconversion after challenge, especially for the BTV-8 inoculated ones, could be explained by residual viral particles from the inoculum at those tissues. Furthermore, BTV detection in tissues at 8 dpi in our study are in agreement with those previously reported [48], which demonstrated both for domestic sheep and goat, that spleen, lymph nodes and lungs are target organs. Moreover, BTV was also detected in the gut-associated lymphoid tissue and liver of goats, as we also found by RT-PCR, RT-qPCR and virus isolation. While domestic sheep and goat showed histopahologic lesions compatible with BT, Spanish ibex did not.

This study demonstrates that: (1) Spanish ibex can be infected with BTV-1 and BTV-8 but is not affected clinically; (2) One single dose of monovalent vaccine prevents BTV viraemia of both BTV-1 and BTV-8. It also suggests that pathogenesis and host immune response may vary among the different BTV strains and that Spanish ibex can contribute to the maintaining of BTV confirmed by viraemia detected until 28 dpi and probably for longer periods. To the authors' knowledge, this is the first study involving two BTV serotypes immunization and experimental infection in Spanish ibex, which may be useful for possible strategies to control BTV transmission from and among wild ruminants.

## Materials and Methods

### Ethics statement

Animals included in the present study were ibex from the Captive Breeding Center of Sierra Nevada (Granada, southern Spain). Permits for vaccination and transport were approved by the Consejería de Medio Ambiente – Junta de Andalucía (Registration number: 1626). Handling procedures and sampling frequency were designed to reduce stress and health risks for subjects, according to European (86/609) and Spanish laws (R.D. 223/1988, R.D.1021/2005), and current guidelines for ethical use of animals in research (2006). The present study was approved by the Ethical and Animal Welfare Committee of the Universitat Autònoma de Barcelona (Permit Number: 4485).

### Vaccination

Fifteen Spanish ibexes (four females and eleven males) one to three years old were distributed in three groups. Four out of the fifteen ibexes were subcutaneously vaccinated with a single dose of 2 mL of BTV-1 inactivated vaccine (Syvazul 1, 10003P; Laboratorios SYVA, León, Spain) on the dorsal region of the neck. Other four ibexes underwent the same handling with BTV-8 (Syvazul 8,10005P, Laboratorios SYVA, León; Spain). The remaining seven ibexes were kept as non vaccinated controls. All ibexes used in the present study were seronegative by ELISA and SNT and RT-PCR negative before BTV vaccination.

### Experimental infection

Twenty-seven days after the vaccination (dpv), the fifteen ibexes were transported to the Biosafety level 3 (BSL3) facilities of the Centre de Recerca en Sanitat Animal (CReSA, Bellaterra, Spain). The four BTV-1 vaccinated and three non vaccinated ibexes were housed in one box (box 1), whereas the four BTV-8 vaccinated ibexes were housed in another box (box 2) with four non vaccinated ibexes. After an adaptation period of five days, all the ibexes except one non vaccinated ibex in box 2, were challenged against BTV-1 (box 1) or BTV-8 (box 2) serotypes with 2 mL of BTV viral suspension in the jugular vein. Viral inocula consisted of infected Vero (African green monkey kidney) E6 culture supernatants of BTV-1/ALG/2006/E6 strain (six passages) with 10^6.5^ TCID_50_/mL (50% tissue culture infective doses) and BTV-8/BEL/2006/E6 strain (five passages) with 10^6.6^ TCID_50_/mL. The viruses were given one passage on embryonated chicken eggs, one passage on baby hamster kidney cells and three (BTV-8) or four (BTV-1) on Vero cells.

Blood samples (with and without EDTA) were collected by jugular puncture, and clinical signs and rectal temperature were measured at days 0, 2, 4, 7, 9, 11, 14, 17, 21, 24 and 28 post-inoculation (dpi). Heparinized blood was collected at 0, 7, 14, 21 and 28 dpi to obtain peripheral blood mononuclear cells (PBMCs). Body weights were measured at 0 dpv (−33 dpi) and at necropsy (8 or 28 dpi). Sera was extracted from whole blood tubes after centrifugation (300× G for 15 minutes) and stored at −20°C. EDTA blood was stored at 4°C until analysis.

At 8 dpi, three non vaccinated ibexes (one inoculated with BTV-1, one with BTV-8 and the one non inoculated ibex) were anesthetized with xylazine (Xilagesic 20%, Laboratorios Calier, 1 mg/kg) and euthanized with an overdose of barbiturate (intravenous infusion of pentobarbital at 100 mg/kg) to study BTV lesions at viraemia peak period. By 28 dpi the remaining twelve ibexes were euthanized using the same protocol. At necropsy, ordinary sampling was performed, including tissue collection (spleen, lung, liver, kidney, bowel, skin, tongue, lip, skin, nasal and oral mucosae, palate, pulmonary artery, heart, epididymis, testicle, urinary bladder, ileum, ileocaecal valve, and mediastinal, mesenteric, axillary and iliac lymph nodes) for BTV RNA detection, BTV isolation and histopathological studies.

### Serology

Sera before vaccination (−33 dpi) and at −5, 0, 2, 4, 7, 9, 11, 14, 17, 21, 24 and 28 dpi were analyzed for the presence of specific antibodies against the BTV major core protein VP7, using a commercial double-antigen ELISA assay (Ingezim BTV DR12.BTV.KO Ingenasa, Spain).

Serotype specific antibodies were detected by means of serum neutralization test (SNT) as described previously [49]. Briefly, serum samples were inactivated at 56 °C for 30 minutes prior to analysis. Sera were diluted 1∶2 to 1∶4096 in microplates (Costar® Cat. N° 3915, Cultek, Madrid, Spain) using MEM Earle (Eagle's minimum essential medium with Earle salts) and mixed with 100 TCID_50%_ of each reference strain (BTV-1 and BTV-8). Samples were tested against both BTV-1 and BTV-8 to determine a possible cross-neutralization of BTV serotypes. Mixtures were incubated for one hour at 37 °C, and 100 µl of a Vero E6 cell suspension in MEM supplemented with 15% foetal bovine serum (FBS; PAA Laboratories GmbH, Austria), 300 µg/l-glutamine/ml, 300 U penicillin/ml and 300 µg streptomycin/ml, were added to a final concentration of 1.5×10^4^/well. The mixture was further incubated for 6 days at 37 °C, plate readings for cytopathic effect (CPE) were done at 4 and 6 days. Developing CPE was compared with control wells containing 100 TCID_50%_ of virus and negative control wells (without virus). Only samples that showed neutralization (absence of CPE) at dilutions ≥1∶4 were considered positive to avoid false positive results from unspecific reactions of sera.

### BTV detection and isolation

Total RNA was extracted from EDTA blood at 0, 2, 4, 7, 9, 11, 14, 17, 21, 24 and 28 dpi and tissue samples from necropsy (8 and 28 dpi) using the Nucleospin® Viral RNA Isolation kit (Macherey –Nagel GmbH & Co, Cultek, Madrid, Spain). All samples were analyzed by RT-PCR and confirmed by real-time quantitative RT-PCR (RT-qPCR). RT-PCR was performed according a procedure previously described [49], [50]. Primers amplified a region of segment 5 (NS1) as previously described [51]. PCR products were visualized by electrophoresis on agarose gel stained with ethidium bromide. RT-qPCR was performed using the primers and the specific probe for segment 5 of BTV described by Toussaint et al. (2007) [52]. Amplification of BTV was carried out using an AgPath-ID™ One-Step RT-PCR kit (Applied Biosystems) in 7500Fast equipment using 2 µl of eluted RNA in a total volume of 20 µl. According to the National BTV Reference Laboratory in Algete (Madrid), reactions were carried out using an amplification program consisting of an initial denaturing step at 95°C for 5 minutes and the following cycling conditions: 1 cycle at 48 °C for 10 minutes, 1 cycle at 95 °C for 10 minutes and 40 cycles at 97°C for 3 seconds and 61°C for 30 seconds. By including serial dilutions (over six orders of magnitude) of a known titrated virus in each RT-qPCR test, estimated titres of each sample could be calculated. The estimated titres could be expressed in the form of an equation of linear regression matching the relation of virus titre against *Ct* values (coefficient of regression: R^2^≥0.99).

BTV isolation was performed from blood and tissue samples by inoculating 500 µL of lysed EDTA blood or tissue supernatants, respectively, onto six well plates of confluent Vero cells. After 90 minutes of incubation at 37 °C, the inoculum was removed and replaced with fresh MEM. Cells were incubated at 37 °C for five days. A second cell passage was done to amplify virus replication and enable final CPE reading as previously described [53].

### Interferon-gamma response in PBMCs

PBMCs from 0, 7, 14, 21 and 28 dpi were isolated being layered on a density gradient (Histopaque d = 1.077; Sigma-Aldrich, Spain) and centrifuged at 350× G for 30 minutes. Trypan blue stain was used to assess cell viability. Cells were re-suspended in RPMI medium (Invitrogen, Spain). Frequencies of BTV-specific interferon-gamma (IFN-γ) secreting cells (SC) in PBMCs were analyzed by an Enzyme linked inmuno spot assay (ELISPOT) using commercial monoclonal antibodies (mAbs) (Bovine IFN-γ AM05875PU-N and AM05867BT-N, Acris, AntibodyBcn, Spain). Briefly, ELISA plates (Costar 3590, Corning, USA) were coated overnight at 4°C with 5 µg/mL of IFN-γ capture antibody (AM05875PU-N) diluted in carbonate–bicarbonate buffer (pH 9.6). Plates were then washed and blocked for 1 hour at 37°C with 150 µl of PBS with 1% of bovine serum albumin. After removal of the blocking solution, 2.5×10^5^ live PBMC were dispensed per well in triplicates and stimulated with phytohaemagglutinin (PHA) (10 µg/ml) (Sigma-Aldrich, Spain) and BTV-1 or BTV-8 strains at 0.04 of multiplicity of infection (moi). The BTV strains were the same used previously at challenge. Non stimulated cells (only RPMI) were kept as background controls. After 20 hours of incubation at 37°C in a 5% CO_2_ atmosphere, cells were removed, and the biotinylated detection antibody (AM05867BT-N) was added at 2.5 µg/mL (50 µL) and incubated for 1 hour at 37°C. The reaction was revealed by sequential incubation of plates with streptavidin-peroxidase at 0.5 µg/mL for 1 hour and insoluble 3,3′,5,5′-Tetramethylbenzidine (TMB; Sigma-Aldrich, Spain). To calculate the BTV-specific frequencies of IFN-γ-SC, counts of spots in non stimulated wells were subtracted from counts in virus-stimulated wells. Frequencies of IFN-γ-SC were expressed as responding cells in 10^6^ PBMCs.

### Haematology

Erythrocytic parameters (RBC, HGB, HTC, MCV, MCH and MCHC), WBC and PLT were determined by a semi-automated haematologic counter (Horiba ABX ABC Vet Hematology Analyzers, Scil Vet abc, Divasa-Farmavic, Spain). Differential leukocyte count was performed by identifying 200 leukocytes on blood smears stained with a commercial Diff-Quick-like stain (Quimica Clínica Aplicada, Spain).

### Statistical analyses

A repeated measures analysis of the variance was performed to detect statistical differences regarding specific BTV antibodies (tested by ELISA and SNT), body temperatures, IFN-γ-SC and haematological parameters, using the PROC MIXED COVTEST procedure of SAS 9.1. (SAS Institute Inc., Cary, NC, USA). The main factor was vaccine (vaccinated or non-vaccinated) and the repeated factor was DPV (day post vaccination). Differences were considered statistically significant when *P*-value<0.05.
